# Leucocytosis in Appendicitis

**Published:** 1902-07-26

**Authors:** 


[1] Lancet, July 12.

### LEUCOCYTOSIS IN APPENDICITIS.

As a result of a careful hæmatological investigation of 20 cases of appendicitis, Dr. Nepean Longridge,[1] senior house physician to St. George's Hospital, comes to much the same conclusions as have been arrived at by Cabot, da Costa, and others. It is not claimed that the leucocyte count will give an absolute and infallible indication of the presence or absence of pus. But such an indication is not, as he truly says, of the most vital importance since pus, *per se*, does not kill. The important thing to know is whether the process which is going on is one of increasing virulence or not, and it is considered that this is a question which can be answered by the blood count, for a progressive increase in the number of leucocytes may be taken as evidence that the inflammatory lesion is developing in severity, and may be reaching the stage of pus formation. But perhaps of even greater importance than mere number is the character of the leucocytes, since "it is found that in almost every case the polymorphonuclear cells are increased out of proportion to the other elements;" a fact which, even when taken alone and apart from any great increase in the absolute number of leucocytes, is of the greatest significance. There are certain conditions, however, in which even in the presence of appendicitis there may be no leucocytosis, namely:—(1) The mild catarrhal variety; (2) fulminating attacks where the resistance of the patient is too feeble to react to the toxæmia; and (3) where an abscess is of some standing, and has become thoroughly walled off.

